# ‘Addressing HPV vaccine hesitancy: unveiling concerns and building trust’ perspectives of adolescent girls and parents in Kisumu County, Kenya

**DOI:** 10.3332/ecancer.2024.1735

**Published:** 2024-08-05

**Authors:** Edwin Onyango Ochomo, Philiph Tonui, Kapten Muthoka, Sayo Amboka, Peter Itsura, Elkanah Omenge Orang’o, Barry Rosen, Patrick Loehrer, Susan Cu-Uvin

**Affiliations:** 1 AMPATH, PO Box 4606 – 30100, Eldoret; 2 KEMRI - RCTP, PO Box 614 – 40100, Kisumu; 3 Moi University - School of Medicine, PO Box 4606 – 30100, Eldoret.; 4 Division of Gynecologic Oncology, Oakland University William Beaumont, Rochester Hills, MI, United States; 5 Indiana University School of Medicine, Indianapolis, IN, USA; 6 Brown University, Providence, RI

**Keywords:** adolescent girls, cervical cancer, HPV vaccine, Kenya, vaccine hesitancy

## Abstract

**Introduction:**

Human papillomavirus (HPV) causes cervical cancer, and HPV vaccination is highly effective in preventing vaccine-targeted HPV infection. However, low HPV vaccination coverage in Kisumu County, Kenya, at about 10% for the first dose, highlights the critical issue of vaccine hesitancy, particularly in low and middle-income countries.

**Methods:**

This study explores the concerns, myths and barriers to HPV vaccine uptake among adolescent girls (aged 10–14) enrolled at human immune-deficiency virus comprehensive care clinics and their parents in Kisumu County. Focused group discussions were conducted with 48 participants.

**Results:**

Content analysis revealed limited knowledge about the HPV vaccine and widespread misconceptions regarding its safety and efficacy. Financial constraints, injection fears and negative clinic experiences emerged as additional barriers.

**Conclusion:**

The findings emphasise the role of effective communication strategies, including engaging parents through written materials and involving them in decision-making, to dispel myths, provide accurate information and encourage HPV vaccination. Collaborative efforts with community stakeholders are crucial to improve vaccine coverage and ultimately reduce the cervical cancer burden.

## Background

Globally, cervical cancer poses a significant threat, with 604,127 cases and 341,831 deaths recorded in 2020 [1]. The burden is particularly high in low- and middle-income countries (LMICs) due to low human papillomavirus (HPV) vaccination rates and limited access to early cervical cancer screening [1]. Human immune-deficiency virus (HIV) prevalence further exacerbates the risk in sub-Saharan Africa (SSA) as it weakens the immune system and reduces vaccine efficacy, especially in women and adolescent girls [2, 3].

HPV vaccination is highly effective, preventing up to 90% of HPV infections associated with cervical cancer [4–7]. However, LMICs face significant challenges, with vaccine coverage among girls in the targeted age group hovering around 15%, far below the global average [8, 9].

In Kenya, the government introduced free HPV vaccination for girls aged 10–14 in 2019 [10, 11]. Despite this initiative, vaccine uptake remains low at only 33% [12]. Kisumu County has a lower HPV vaccination coverage at only 10% as of the end of quarter four of 2022 [13]. Vaccine hesitancy is a major barrier, hindering efforts to protect young girls from cervical cancer. To address this, understanding the concerns and misconceptions surrounding the vaccine is crucial. This study aimed to explore these issues through discussions with adolescent girls and their guardians in two public health facilities within Kisumu County, Western Kenya.

## Methods

### Study area

The study was conducted in Kisumu County, Kenya, involving adolescent girls aged 10–14 years attending the HIV comprehensive care clinics (CCCs) at Kisumu County Referral Hospital and Lumumba Sub-County Hospital. These are the primary HIV care centres in the county, which has a total of seven sub-counties and ranks third in HIV prevalence nationally (15.6%) according to the KENPHIA report [14].

## Study design

This qualitative sub-study was part of a larger pilot initiative exploring the use of text messages to address HPV vaccine hesitancy. Focused group discussions (FGDs) were conducted separately with adolescent girls and their parents (of both vaccinated and unvaccinated girls) to understand their perspectives on the HPV vaccine. The adolescent girls’ sessions were further divided into vaccinated and unvaccinated groups.

### Sampling procedure

Purposive sampling was employed to recruit participants willing and able to discuss their experiences with HPV vaccination. A total of 24 participants were selected, including adolescents and parents, to ensure a rich and varied understanding of the topic and reach saturation. Trained qualitative research interviewers at the healthcare facilities facilitated the FGDs.

### Data analysis

Audio recordings of the FGDs were transcribed verbatim and translated into English for analysis. NVIVO software was used to conduct content analysis, identifying and grouping related themes.

## Results

Four FGD sessions were held between May 5th and June 3rd, 2023, at the two participating health facilities. Separate sessions were conducted for parents and adolescent girls, with further divisions for vaccinated and unvaccinated girls. Each FGD consisted of eight participants and explored topics such as HPV vaccine knowledge, cervical cancer awareness, common myths surrounding the vaccine (e.g., safety concerns and fertility impacts) and factors influencing vaccine access.

### Vaccine knowledge

Respondents demonstrated awareness of at least one vaccine, with a significant number mentioning the corona virus disease-2019 (COVID-19) vaccine. Notably, one unvaccinated adolescent girl remarked, ‘**There is a COVID-19 vaccine’,** while a vaccinated girl mentioned, **Just any? I know corona vaccine’.** During the first parent FGD, a participant explained**, ‘These are protection given to people before they fall sick to prevent diseases like COVID vaccine’,** similarly, in the second parent FGD, a parent mentioned the ‘**COVID vaccine’.** Notably, several respondents also recognised the HPV vaccine as a preventive measure against cervical cancer. They explicitly mentioned phrases like **‘HPV vaccine’** and **‘There are several vaccines to protect children against diseases like this one against cervical cancer.’ (***Unvaccinated adolescent girl)****.*** Moreover, participants understood that the HPV vaccine’s purpose was to shield girls from the development of cervical cancer, with one saying, **‘Cervical cancer vaccine is that vaccine that you are given to prevent cervical cancer from getting into your body.’** (*Vaccinated adolescent girl)*

### Eligible population

The majority of respondents were aware that the recommended age range for HPV vaccination is between 9 and 14 years old. They generally agreed that this age range is appropriate for vaccination. One parent emphasised, ‘***I think the adolescent stage is very vital and it is at this point that they should get such things as this vaccine. Just before, they start engaging in intercourse***’. Another parent from FGD 1 shared similar sentiments stating that, ‘I will co***ncur with the person who has just talked, this adolescent stage is very risky because these girls start becoming active for sex and even you as a parent, they will disturb you. So, it is a good stage to have these girls get the vaccine’***. Other comments supporting vaccination of adolescents included, ‘***I think the adolescent stage is very vital and it is at this point that they should get such things as this vaccine. Just before they start engaging in intercourse’*** and that ‘***that age group should be vaccinated because at that age, there are various diseases which infect them’.*** These were sentiments from the parents.

However, a few parents expressed concerns about the age group’s limitation, suggesting that some eligible individuals might be excluded. A parent noted that, ‘***The starting age is just okay but what they can reconsider is the maximum age of getting the vaccine. If they can increase it a bit since sometimes it was brought when some people are already beyond that age and missed out,’*** another parent suggested, ‘***they should have increased the age limit a bit. Since they have put it at 10–18 years, they should have increased it to maybe 25 or 30 years. That will really help these children but they are still not very old. You may get a child who is already over 18 years but is still in school and therefore still a child but because of the age, they cannot be vaccinated’***.

### Vaccine availability and access points

A significant number of participants noted that the HPV vaccine is accessible at public hospitals and is administered during vaccination outreach programs in schools. One adolescent responded that, ‘***Yes, I can get it from the hospital, school and even in church***…’ while another one said, ‘***In the hospital and school’***.

### Challenges hindering HPV vaccine uptake

Various barriers to receiving the HPV vaccine were identified, primarily revolving around concerns about vaccine safety and efficacy, uncertainty about vaccine access and healthcare provider attitudes. One unvaccinated adolescent girl expressed, ***‘For me, I have seen it on TV but I did not see them talk about the benefits of the vaccine***.’ Similar sentiments were echoed by another unvaccinated girl saying, ‘***They also never tell us about these side effects, so when those who have been vaccinated talk about the side effects, even you who would you believe? So, they should explain these things…’*** and*** ‘Yes but they never talk about how to go about it and from which facilities to get it from.’***

Participants also raised concerns about the inadequacy of posters in health facilities: One vaccinated adolescent girl noted, ***‘I have never seen it in the hospital. Maybe it is there but they have not brought it to the adolescent centre where we normally get our drugs from.’***

Not knowing where to obtain the vaccine was cited as a barrier by those who had not yet been vaccinated, an unvaccinated adolescent girl noted that ‘***I didn’t know where to get the vaccine.’*** And another one explained, ‘***I haven’t because I don’t know where those people are.’***

Financial constraints hindered accessing vaccination clinics as expressed by one unvaccinated adolescent: ***‘You see for us adolescents if you are to go and look for the vaccine you have to look for money first and we don’t have the money.’***

Fear of the injection was also pointed out as a barrier to getting the vaccine: ‘***First, I fear injections…***’ Unvaccinated adolescent girl. One vaccinated adolescent girl also commented that, ‘***maybe they also fear or they have seen how people react after being vaccinated.***’ Another unvaccinated adolescent girl lamented that, ‘***future but that injection looks very painful, why can’t they bring tablets we can swallow?***’ Similarly, one parent from the second FGD noted ‘***There are also girls who instil fear in people after they have gone. They tell you not to go because it is a painful process.***’

Health system factors, particularly staff attitudes, were also identified as a hindrance, one vaccinated adolescent girl explained: ***‘…It also depends on the nurse who is giving the injection. Like for us the nurse who was vaccinating us was very rude, she would tell you to sit down and get the vaccine and if you don’t want you can leave.’*** another vaccinated girl retorted, ‘***Yes, so it depends on the nurse, some are very rude and harsh, you can even leave before it is your turn to get the vaccine.’*** Finally, time constraints due to competing tasks for both the parents and the school going adolescent girls further posed difficulties ***‘…once you tell your parent, mother or father, to take you to the hospital, they will say they don’t have time,’*** *Unvaccinated adolescent girl*, and ***‘I have been busy with school and they didn’t bring it to the school.’*** *another unvaccinated girl.*

### Myths and misconceptions surrounding the HPV vaccine

Insufficient dissemination of information to the community has allowed myths and fears to prevail and derail the efforts to improve vaccine coverage: ‘***I think the information has not really reached the people well, there are so many fears out there which need to be addressed.’*** *A parent in FGD 1.*

Within the communities where the respondents reside, numerous myths and misconceptions regarding the HPV vaccine exist. These include notions that the vaccine causes infertility in adolescent girls, induces paralysis and leads to unexplained illnesses. One parent in FGD 1 stated, ‘***I have heard people oppose saying that those girls who have been vaccinated will not be able to give birth’*** while a parent in FGD 2 shared ‘***I have heard people talking about the vaccine and they say that they still don’t know about the vaccine well, some say this vaccine is meant to spoil the kids.’*** An unvaccinated adolescent girl mentioned that, ***‘Some people say that if you get vaccinated you will not get a child in future. That you can’t get pregnant in future.’*** Another unvaccinated adolescent girl shared that, ***‘Some people say it is bad and brought by the whites to sterilise Africans.’***

Additionally, concerns arose about the vaccine’s impact on the future of adolescent girls’ sexual health, one parent from FGD 2 explained that ‘***Some people also lie that the vaccine interferes with the feelings such that the children will not have any feelings and can even fail to get married.’***

Some of the respondents claimed that the vaccine causes unexplained sickness, which led to them shying away from getting the vaccine to keep themselves safe, one unvaccinated adolescent inquired, ***‘Some also say that when you get vaccinated you always feel sick all the time, so I don’t know if it is true...’*** and another one added that ‘…***you will keep getting sick from time to time’***

Furthermore, unfounded beliefs about the vaccine causing paralysis emerged, a vaccinated adolescent girl explained: **‘I heard* people say that if you get vaccinated you will get paralysed on the hands and you will keep getting sick from time to time.’***

However, a genuine concern regarding promiscuity among vaccinated adolescent girls was voiced: ‘It*** is also said that once the girls get vaccinated they will not fear getting infection, they will just be sleeping with boys and men.’*** a parent from FGD 2. This was well responded to by one of the parents in FGD2 who pointed out the need for parents to provide guidance to the girls after getting the vaccine: ***‘But this vaccine is only protecting the girls against HPV but not other infections like syphilis and even pregnancy, so as parents we should teach our daughters on how to keep safe***.’

### Enabling factors for HPV vaccination uptake

Vaccinated adolescent girls and parents who consented for their daughters to receive the vaccine highlighted several factors that motivated their decision. Effective vaccine communication and accurate information dissemination played a crucial role, a parent from FGD 2 explained, ***‘For me, my child was given a piece of paper, for those who wanted their daughters to be vaccinated they filled the form but those who did not want the vaccine did not fill the form. So I filled the form because I had already heard about the vaccine being advertised on the radio’*** while one vaccinated girls also supported the sentiments, ***‘…before I got the right information never knew about how important it was but after being trained at DREAM GIRL (DREAM is a girls’ mentorship program for adolescents), I got to know how important the vaccine is and got vaccinated.’***

Parental approval and consent were pivotal facilitators, as reported by one vaccinated adolescent girl: ‘***No, we were given forms to take to our parents, so when they came to the school they asked how many people their parents agreed that they could get the vaccine. So they went to each class to vaccinate those whose parents had agreed to have the vaccine.’*** Another vaccinated adolescent girl also explained, ***‘…he asked me if he can ask my mother to allow me to get the vaccine then I told him to just ask although if it is the corona vaccine then I don’t want and if it is that one which makes the hand become heavy, I also don’t want. That’s when my mother agreed, and I got vaccinated.’***

Another parent’s motivation stemmed from the awareness of cervical cancer’s impact, ‘***There is a day I went to the hospital and I heard about the vaccine. I was happy about what was being said especially that last year but one and even last year, there were many deaths among women because of cervical cancer. Therefore, when I heard about this, I was imagining that I may be having cancer already but I would not want my child to get cancer. For me I already came for screening and found that I do not have it so I also wanted to protect my child, so I took her for the first dose and even the second one.’*** A parent from FGD 2.

Moreover, the perception of the vaccine’s efficacy in preventing future cervical cancer was a driving force. One vaccinated girl explained her reason for getting vaccinated, ‘***I got the vaccine to prevent cervical cancer in future’*** while a parent from FGD 1 noted that ‘***I think there is an age of adolescence when these children are very stubborn, they want to do this and that so if the child has been vaccinated they are protected from infection.’***

### Community’s role in enhancing HPV vaccine uptake

The community’s involvement is pivotal in ensuring optimal vaccine coverage. One approach involves enlightening eligible adolescent girls about the vaccine’s importance and encouraging them to seek vaccination: a parent from FGD 1, ‘***we will talk to them and send them to the hospital to get the vaccine***.’ This was supported by another parent from FGD 1 saying, ‘***Once we know about the vaccine, we will talk to them and send them to the hospital to get the vaccine.’*** Another way is for the community to encourage the girls to complete the recommended doses: ‘***So like mine came after getting the first dose, so all I can do is to ensure that they get the remaining doses***.’ a parent from FGD 2.

Creating a conducive environment was also emphasised as a community-driven strategy to encourage vaccine uptake, ‘***You can also provide a conducive environment that will encourage the child to go for the vaccine by not quarrelling in case they get vaccinated.***’ A parent from FGD 1.

### Strategies to overcome HPV vaccine hesitancy

Respondents proposed a range of strategies to effectively address HPV vaccine hesitancy:

Countering the myths with facts and addressing the fears held by the communities about the HPV vaccine: ‘***I think the information has not really reached the people well, there are so many fears out there which need to be addressed.’*** A parent from FGD 1, and ‘***…most importantly they should teach people about the vaccine itself.***’ Another parent from FGD 1

Second, holding dialogue sessions with the communities which allows for feedback and addressing of specific concerns and questions of the community, a parent from FGD 2 exclaimed: ‘***My concern is the way this information is being shared; those posters and advertisements on TV and radio do not give us the opportunity to ask questions.***’

Third, to involve the targeted adolescent girls in the conversations about being vaccinated and sharing with them the vaccine information with them directly: ‘***I also feel that since this vaccine is meant for girls, they should also be talked to. For example, these teenagers can be very stubborn, you will send her to go and get the vaccine but not reach the clinic, so if they are talked to and understand it will even be asking us as parents to allow them to get the vaccine.’*** *A parent from FGD 1.*

Furthermore, the healthcare providers need to be empowered to possess accurate information to educate clients and address their questions. ***‘The other concern is with the clinics, they do not seem to be ready to share the information when asked. So you are left wondering whether they don’t know or they just don’t want to share.’*** *A parent from FGD 1.*

Other strategies that can be explored include; availing information, education and communication materials to the target population, one unvaccinated adolescent girl suggested, ‘***They can print booklets with information on cervical cancer and the vaccine.’***, ensuring the vaccine is available at no cost: ***‘If the vaccine can be available at no cost since there are places you will go and they will ask you for money before they can vaccinate you.’*** *Unvaccinated adolescent girl*. The government can also take the vaccine to the schools and to the community: *Unvaccinated adolescent girl,* ‘***The government can send the vaccinators to the schools; you see the schools have hundreds of girls who are eligible for vaccination yet at home parents might be busy and not get time to take the girls to the clinic for the vaccine’*** and also one vaccinated girl suggested, ***‘Since I live far from the facility, if they can come to the community like they do for children then it can save us on transport cost.’***, utilising social media to reach out to the adolescent girls: ***‘Use of social media to share the information because young people now like social media.’*** *Unvaccinated adolescent girl.*

## Discussion

Addressing the causes of vaccine hesitancy is crucial to achieving the World Health Organisation target of vaccinating at least 90% of eligible girls by age 15 [15]. Findings from the FGDs revealed that there was relatively good knowledge on vaccines in general and who should get the vaccine. However, the respondents did not have a good grasp of the HPV vaccine, coupled with a lot of myths and misconceptions leading to vaccine hesitancy.

The primary obstacle to vaccinating eligible girls centred on inadequate information regarding the vaccine’s safety and effectiveness. Moreover, unfamiliarity with vaccination centres and, to some extent, negative staff attitudes played a role. Importantly, individuals who received accurate and well-delivered information about the vaccine were more inclined to accept it. Additionally, the community emerged as a pivotal factor in addressing vaccine hesitancy.

Having a comprehensive understanding of the different types of available vaccines serves as an indicator of effective overall vaccine awareness. This knowledge can contribute to countering vaccine hesitancy and fostering positive vaccination choices [16]. Despite of relatively strong grasp of vaccines in general, only a small number of respondents could identify the HPV vaccine to be among the vaccines they were familiar with. Similar findings were reported in Brazil, where respondents did not know about the HPV vaccine [17]. A comparable low understanding of the HPV vaccine was reported among parents in Texas [18]. This lack of knowledge can be attributed to inadequate sensitisation by the public health department and the bias towards other vaccines such as polio, Bacillus Calmette-Guerin and measles which are already in the vaccination schedule at the maternal and child clinics.

In the absence of proper communication channels to inform the communities about the vaccine and address the concerns, the misinformation reigns supreme causing vaccine hesitancy. Our findings identified several barriers to vaccination including, inadequate information about the HPV vaccine and infection, uncertainty about vaccination locations, financial constraints, fear of injections and negative staff attitudes. Similar findings were reported in Austria and Uganda where key barriers to vaccine uptake were inadequate information about HPV infection and HPV vaccine, and concerns about HPV vaccine efficacy and safety [19, 20]. Moreover, an analysis of the HPV vaccine hesitancy trends between 2010 and 2020 indicates that lack of information is a major barrier to getting vaccinated [21]. This is an indication that the current HPV vaccination campaigns are yet to properly deal with the myths widely held by the communities.

The myths and misconceptions surrounding the safety and effectiveness of the HPV vaccine were prominent, as seen in previous research which also noted myths of the vaccine stimulating early sexual activity [22]. Similar findings emerged in our current study where both the parents and the adolescent girls questioned the safety and effectiveness of the vaccine with concerns of the vaccine causing infertility and unknown illnesses were noted.

However, the most notable facilitators to the adolescent girls being vaccinated were proper communication about the vaccine to the parents and guardians. Where parents were given written communication from schools and those engaged by the healthcare providers to allow their daughters get the vaccine allowed their daughters get the vaccine. These findings are similar to those reported in a systematic review looking at barriers and facilitators to HPV vaccination in SSA which reported that proper communication and sharing information about the vaccine facilitated vaccine uptake [23]. Through clear communication and information sharing, either by community dialogue and meetings or the distribution of leaflets and bronchus, dispelling myths and misconceptions becomes achievable, ultimately diminishing their impact on vaccine acceptance. Furthermore, collaboration with community stakeholders like community leaders, religious figures and political leaders, by enlightening them and ensuring that they are well informed has also been recommended as an effective strategy to address vaccine hesitancy, where they are used to sensitise their communities about the vaccines [24]. These leaders are respected in their communities and are able to influence decisions in the local communities and even policies at the national level. Current communication strategies utilising posters and audio-visual media seem not be provide assurance about the safety and effectiveness of the vaccine since they do not provide an opportunity for questions to be answered and clarifications to be sought.

## Conclusion

Vaccine hesitancy towards the HPV vaccine primarily is largely driven by inadequate communication strategies employed by key players and stakeholders within the health sector. Unfortunately, the perspectives and opinions of the very population these strategies aim to influence have often been disregarded. This lack of engagement of the target population has led to an incomplete understanding of their concerns. Engaging both the parents and guardians to understand their concerns has the potential to overcome the barriers to vaccine uptake. Similarly, working collaboratively with parents and adolescents to develop communication strategies that resonate with them offers the potential for broader application and implementation. These insights underscore the critical importance of tailored communication and collaboration in shaping successful public health interventions and achieving higher HPV vaccine coverage rates.

## Limitation

The study was limited to only the adolescents enrolled at the CCC of the two sampled facilities and their parents. This provided a small sample size which limits the generalisability of the study findings.

## List of abbreviations

CCC, Comprehensive care clinic; COVID-19, Coronavirus disease-2019; FGD, Focused group discussion; HIV, Human immune-deficiency virus; HPV, Human papillomavirus; LMIC, Low-and-middle income countries; SSA, Sub-Saharan Africa.

## Conflicts of interest

The authors declare that they have no competing interests.

## Funding

This study was supported by ‘The East Africa Consortium for HPV and Cervical Cancer in Women living with HIV/AIDS’, Grant number: 5U54CA254518-03.

## Consent for publication

Not applicable.

## Ethics approval and consent to participate

The study obtained ethical approval from the Moi Teaching and Referral Hospital/Moi University’s Institutional Research and Ethics Committee (REF: IREC 346/2022) and research permit issued by the National Commission For Science, Technology and Innovation (No. NACOSTI/P/23/24291). Before enrolment of the adolescent girls, verbal informed consent was obtained from the parent and the adolescent girls gave written informed assent. For the parents, written informed consent was obtained before enrolment.

## Availability of data and materials

The datasets used and/or analysed during the current study are available from the corresponding author on reasonable request.

## Authors’ contributions

OEO designed and carried out the data collection in the field and participated in the drafting of the manuscript. PT, KM, EOO and PI made substantial contributions to the design and interpretation of the data. SA reviewed and analysed the data. OEO, KM, SA, PT, PI, EOO, BR, PL and SC were involved in revising the manuscript critically for important intellectual content. They also gave the final approval of the version to be published and have agreed to be accountable for all aspects of this work. All authors read and approved the final manuscript.
